# A systematic review and meta-regression of exogenous ketone infusion rates and resulting ketosis—A tool for clinicians and researchers

**DOI:** 10.3389/fphys.2023.1202186

**Published:** 2023-06-28

**Authors:** Kristi L. Storoschuk, Thomas R. Wood, Brianna J. Stubbs

**Affiliations:** ^1^ School of Kinesiology and Health Studies, Queen’s University, Kingston, ON, Canada; ^2^ Department of Pediatrics, University of Washington, Washington, WA, United States; ^3^ Institute for Human and Machine Cognition, Pensacola, FL, United States; ^4^ Buck Institute for Research on Aging, Novato, CA, United States

**Keywords:** ketones (plasma), beta-hydroxybutyrate (BHB), infusion, pharmacokinetics, exogenous ketone

## Abstract

**Introduction:** Ketone bodies such as beta-hydroxybutyrate (BHB) have pleiotropic functional benefits as fuel and signaling metabolites and may have multiple clinical applications. An alternative to inducing ketosis by dietary modification is intravenous delivery of exogenous sources of ketones. It is unknown whether there is a strong relationship between BHB infusion rate and blood BHB concentrations in the published literature; this information is vital for clinical studies investigating therapeutic effects of ketosis. This systematic review aimed to aggregate available data and address this gap.

**Methods:** The PubMed and EMBASE databases were searched, and data were extracted from 23 manuscripts where BHB was infused and maximum and/or steady state BHB levels assessed. Infusion rate was adjusted when racemic BHB was infused but only D-BHB was measured.

**Results:** Using a random effects meta-regression, strong linear relationships between BHB infusion rate and maximal (y = 0.060 + 0.870x, *R* ^2^ = 87.2%, *p* < 0.0001) and steady state (y = −0.022 + 0.849x, *R* ^2^ = 86.9%, *p* < 0.0001) blood BHB concentrations were found. Sensitivity analysis found this relationship was stronger when studies in non-healthy populations were excluded (y = 0.059 + 0.831x, *R* ^2^ = 96.3%, *p* < 0.0001).

**Conclusion:** There is a strong relationship between BHB infusion rate and blood BHB concentrations; the regressions described here can be used by clinicians or researchers to determine ketone delivery required for a target blood concentration.

## 1 Introduction

In human metabolism, the term “ketone bodies” (commonly referred to as “ketones”) refers to beta-hydroxybutyrate (BHB), acetoacetate and acetone (Robinson and Williamson, 1980). “Ketosis” refers to the accumulation of ketones to reach detectable concentrations in the blood. Ketones are increasingly appreciated to function as both fuels and signaling metabolites (Newman and Verdin, 2017), with multiple possible applications in health and disease (Puchalska and Crawford, 2021). Blood ketone concentrations can be increased by endogenous or exogenous interventions. Endogenous interventions include adherence to a ketogenic diet (low in carbohydrate, moderate in protein and high in fat), starvation, caloric restriction, prolonged exercise, or voluntary fasting (Cahill et al., 1966; Koeslag et al., 1980; Hallberg et al., 2018). In these states, low glucose and insulin concentrations leads to an increase in circulating fatty acids, which are metabolized by the liver into ketones (Robinson and Williamson, 1980). A healthy adult can produce ∼150 g of ketones during a period of prolonged fasting (Reichard et al., 1974), reaching a “physiological” concentration of blood BHB of 5–7 mM (Cahill, 1970).

Exogenous methods of inducing ketosis include oral or intravenous (IV) BHB delivery (Stubbs et al., 2017; White et al., 2021). BHB or BHB precursors are the most commonly administered ketone moiety as BHB is the ketone body found in circulation in the greatest abundance (Laffel, 1999), is more stable than acetoacetate and acetone (Fritzsche et al., 2001), and therefore easier to formulate. The IV route typically utilizes a mineral salt of BHB (White et al., 2021), in either a racemic mixture of D-BHB and ʟ-BHB isoforms, or enantiomerically pure, non-racemic salts of the “physiological” D-BHB isoform. IV infusions of BHB have been used extensively in research settings to investigate metabolic function; however, there are possible translational applications for acute in-patient conditions affecting the heart and brain, which both avidly oxidize ketones [summarized in a recent systematic review by White et al. (2021)]. Some research indicates that there could be a threshold or therapeutic window for BHB concentrations associated with various functional benefits. For example, Nielsen et al. (2019) found that cardiac output increased with increasing BHB concentration during an IV infusion, and Fortier et al. (2021) found that cognitive function improved with increasing BHB concentration with oral ketone supplementation. Therefore, precise control of blood BHB concentration could be important to optimize efficacy of exogenous ketone interventions.

Whilst the use of IV BHB in research has been documented since the 1960s (Balasse and Ooms, 1968), the relationship between infusion rate and resulting blood BHB concentrations in the literature has not been closely examined. Comparison of data between studies is made complicated by methodological variations, for example, the study of diseased vs. healthy populations that may vary in their metabolism of ketones, lack of standardization of BHB infusion units by mass vs. moles, infusion of racemic vs. non-racemic BHB salts, and use of differing measurement methods for blood BHB concentrations. Whilst mass spectrometry-based methods quantify total BHB, detecting both D-BHB and ʟ-BHB isoforms, the most common quantification technique is an enzymatic method that is specific to the D-BHB isoform which does not detect the ʟ-BHB isoform delivered in a racemic salt infusion. Therefore, either infusion rate or measured concentration should be adjusted to account for this.

To better inform selection of BHB delivery rates to achieve targeted blood BHB concentrations for research and clinical applications, this systematic review aimed to aggregate and standardize the available literature and determine the relationship between differing IV BHB infusion rates and blood BHB concentrations in any type of clinical study of healthy and non-healthy adults. We hypothesized that there would be a strong, linear relationship between infusion rate of BHB and the blood BHB concentrations, allowing the calculation of a regression that could be used to determine the expected rate of BHB delivery required to achieve a target blood BHB concentration.

## 2 Methods

This systematic review was conducted in accordance with the Preferred Reporting Items for Systematic Reviews and Meta-Analyses (PRISMA) checklist (Moher et al., 2009) (See Supplementary Information) and was registered on Open Science Framework (Storoschuk and Stubbs, 2023). The software program, Covidence (Veritas Health Innovation, Melbourne, Australia), was used during all stages of study selection and data extraction.

### 2.1 Eligibility criteria

Studies were included for the final analyses if they met all of the following inclusion criteria: 1) used human adult subjects with no limitations (above 18 years of age), 2) administered BHB via IV-infusion at rest, 3) reported BHB concentrations from venous or capillary sampling, or 4) reported a maximal BHB concentration for a given infusion rate. Studies were excluded if they: 1) did not meet all of the inclusion criteria, 2) not enough data provided to determine infusion rates (e.g., body weight), 3) were not in English, 4), the full-text was unavailable after an exhaustive search, or 5) was not an original research article.

### 2.2 Literature search and study selection

The systematic searches were conducted using the online databases, PubMed and EMBASE (Ovid) on 29 June 2022. In addition to the electronic database search, studies found by any of the authors that were not captured in the initial literature search but that appeared to meet the inclusion criteria were included as cross-sectional references. The searches included these primary search terms: BHB, beta-hydroxybutyrate, ketone body, IV-infusion, and intravenous. The search terms were adapted to each database. Synonyms for BHB and IV-infusion were separately combined with “OR” and the search was conducted using each list of synonyms combined with “AND”, using the “human” filter. The titles and abstracts were extracted from both database searches and uploaded into Covidence where duplicates were automatically removed.

Two reviewers (KLS and BJS) independently screened titles and abstracts according to the inclusion/exclusion criteria. In the case of conflicts, both reviewers met to discuss their study selection decisions until they reached a mutual decision. Studies that were included on the basis of title and abstract advanced to full-text screening where the same process was followed (KLS and BJS review independent, resolve conflicts together). A reason for exclusion was provided during full-text screening. The final analyses were conducted using studies that advanced through both levels of screening.

### 2.3 Data extraction

Data were extracted independently by a single reviewer (BJS) and confirmed by a second reviewer (KLS) to verify correct data extraction. Information pertaining to the BHB infusion was extracted, including the compound infused, isomer of BHB measured, BHB measurement method, priming dose and continuous dose used of BHB IV-infusion. Infusion rates were standardized to mg/kg/min. Means and error (as standard error, standard deviation, or 95% CI) for BHB concentrations were extracted either by directly extracting from tables/text or by using the data extraction tool, WebPlotDigitizer (WebPlotDigitizer, Pacifica, CA, United States), when data were only reported in figures. The maximum BHB (mean and error) concentration was extracted from all studies. Steady state BHB concentrations were extracted either directly as reported or based on the time course values for infusions >30 min. Steady state was considered as the median time point after BHB concentrations reached 15% of the recorded maximal value, the earlier time point was used when there was an odd number of time points. All measured BHB concentrations were standardized to mM. Participant population, number of participants, and any co-interventions were also extracted. When the BHB formulation infused was racemic (i.e., D/ʟ-BHB), but the method for measuring BHB concentration only measured D-BHB (enzymatic method), the infusion rate was divided by two, as most racemic mixtures are expected to be a 50/50 enrichment of both enantiomers.

### 2.4 Data synthesis and statistical analysis

Data were harmonized by converting standard deviation (SD), standard error (SE), or 95% CI to variance. Linear random effects meta-regressions of continuous infusion dose *versus* maximum or steady state blood BHB concentrations were performed using the metafor package in R. A meta-regression is a statistical model used to determine correlations using multiple datasets (Thompson and Higgins, 2002; Willis and Riley, 2017). Studies that included multiple infusion doses with associated BHB measures were included as separate data points. Results were reported as the associated regression equation, I^2^ (percentage of the unaccounted variability attributable to residual heterogeneity), tau (standard deviation of the effect size), *R* ^2^ (expressed as percentage of variance in circulating BHB explained by BHB infusion dose), and *p*-value for the estimate of the relationship between infused and measured BHB. Graphs were plotted displaying the regression output, where the diameter of the point for each study was proportional to the inverse of the variance. As the presence of a disease such as Type 1 Diabetes, Type 2 Diabetes or Heart Failure might alter ketone metabolism, additional sensitivity analyses were performed with these studies removed. All analyses were performed using R version 4.1.2 in the RStudio environment.

## 3 Results

**FIGURE 1:**
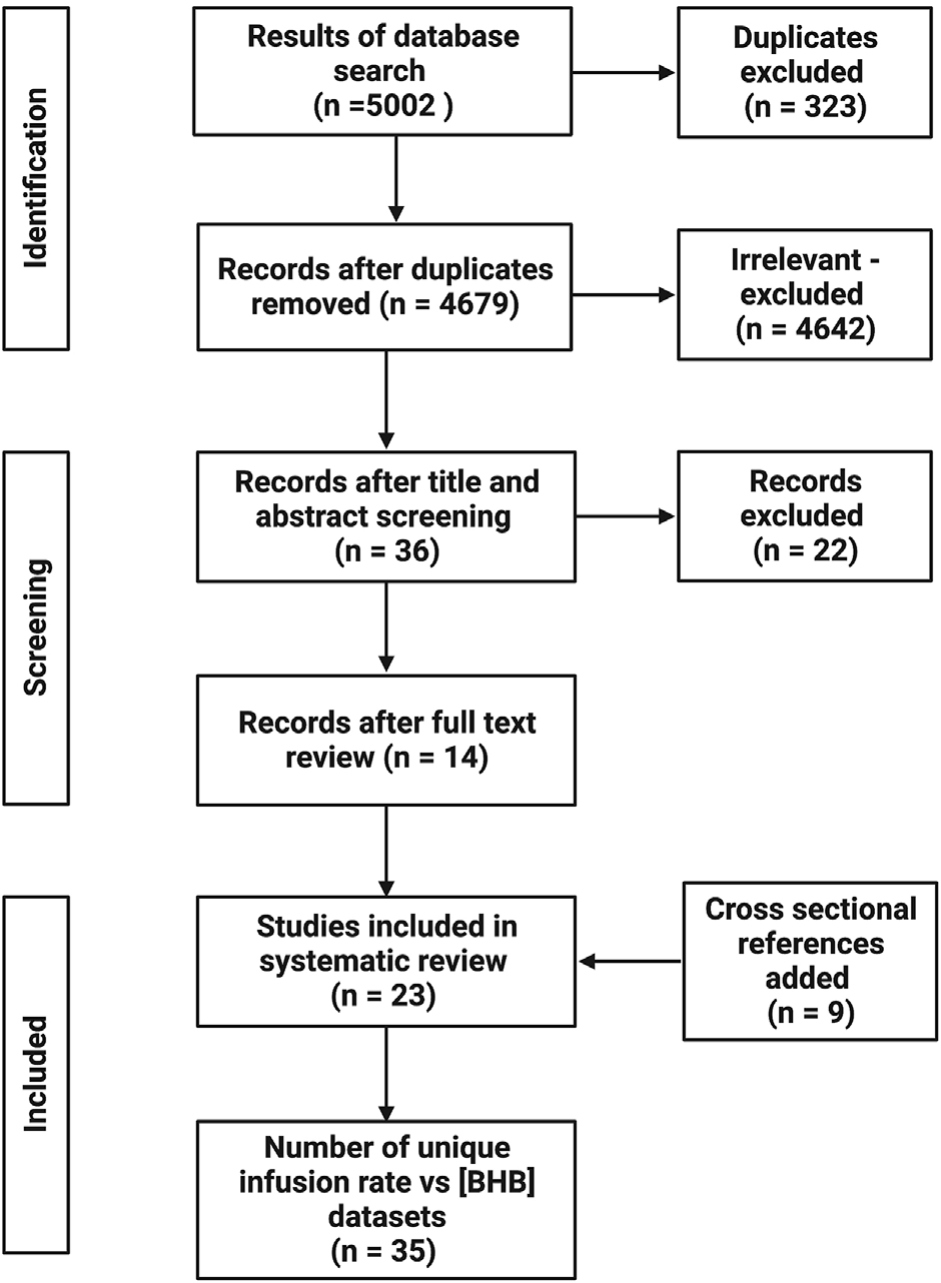
Flow diagram of study selection process. Created with BioRender.com.

The systematic database search retrieved 5002 potentially relevant papers, which was reduced to 4684 following removing duplicates. After title and abstract screening, 36 papers remained for full text screening; 14 of these were included. A further 9 papers were added as cross-sectional references, therefore 23 papers were included in the systematic review. A total of 35 unique infusion rate vs. BHB concentration datasets were extracted, given that 8 papers reported multiple datasets (2 datasets = 5; 3 datasets = 2; 4 datasets = 1). A flowchart of the study selection process is presented in Figure 1.

A summary of study characteristics is presented in Table 1. The majority of papers (*n* = 21/23) infused racemic BHB, rather than enantiomerically pure D-BHB (*n* = 2/23). The most common measurement method for BHB was the enzymatic method, that is, specific to D-BHB (*n* = 18/23); infusion rates were adjusted where racemic BHB was infused but only D-BHB was measured (*n* = 17/23). Several studies included data collected in disease populations (*n* = 10/35 datasets). Many datasets reported both BHB-C_max_ and BHB-C_ss_ (*n* = 23/35), *n* = 8/35 and *n* = 4/35 reported only reported BHB-C_max_ or BHB-C_ss_ respectively. BHB data were presented solely as a time course series in *n* = 14/23 papers; *n* = 7/23 papers gave isolated BHB data in the text or a table and *n* = 2/23 papers included datasets in both formats.

**TABLE 1:** Summary of study characteristics, data fields and datasets that were extracted from studies included in the systematic review.

Author	Study design	Population	Co-conditions	No. Subjects	Compound infused	Priming D-BHB dose (mg/kg/min)	Continuous D-BHB dose (mg/kg/min)	Infusion duration (minutes)	BHB measured	BHB C_max_ (mM)	BHB C_ss_ (mM)	Time course (Y/N)
Sherwin et al. (1976)	NR, CO, PLA+	HA	IV glucose infusion	6	Na-D/ʟ-BHB	N/A	1.5	270	D-BHB	0.98	0.92	Y
		HA	N/A	12	Na-D/ʟ-BHB	3	1.5	180	D-BHB	N/A	0.82	N
		T1D	N/A	7	Na-D/ʟ-BHB	3	1.5	180	D-BHB	N/A	1.67	N
Frolund et al. (1980)	R, CO, PLA+	HA	Hypoglycemia	6	Na-D/ʟ-BHB	4^a^	2	105	D-BHB	0.54	0.51	Y
		HA	Hypoglycemia	3	Na-D/ʟ-BHB	13^b^	6.5	105	D-BHB	5.82	5.49	Y
Christensen et al. (1986)	1A, PLA-	HA	N/A	7	Na-D/ʟ-BHB	N/A	4.15	60	D-BHB	1.94	1.81	Y
Desir et al. (1986)	NR, CO, PLA-	HA	Acidemia	7	Na-D/ʟ-BHB	2.6	0.52	180	D-BHB	0.38	0.28	Y
	NR, CO, PLA-	HA	Acidemia	4	Na-D/ʟ-BHB	2.6	0.52	180	D-BHB	0.39	0.2	Y
Nair et al. (1988)	NR, CO, PLA+	HA	N/A	13	Na-D/ʟ-BHB	2	0.65	480	D-BHB	2.46	2.27	Y
Crowe et al. (1989)	NR, CO, PLA-	HA	Pre-surgery	6	Na-D/ʟ-BHB	3	1.5	140	D-BHB	0.69	N/A	N
	NR, CO, PLA-	HA	Post-surgery	6	Na-D/ʟ-BHB	3	1.5	140	D-BHB	0.62	N/A	N
Moller et al. (1990a)	R, CO, PLA-	T1D	Euglycemia	5	Na-D/ʟ-BHB	1.6	0.78	120	D-BHB	0.8	0.77	Y
		T1D	Hyperglycemia	5	Na-D/ʟ-BHB	1.6	0.78	120	D-BHB	0.66	0.62	Y
Moller et al. (1990b)	R, CO, PLA+	T1D	Hyperinsulinemic euglycemic clamp	6	Na-D/ʟ-BHB	1.6	0.78	200	D-BHB	0.9	0.84	Y
Amiel et al. (1991)	NR, CO, PLA+	HA	Hypoglycemia	6	Na-D/ʟ-BHB	6	3	260	D-BHB	N/A	0.58	N
Chiolero et al. (1993)	NR, CO, PLA+	HA	N/A	6	Na-D/ʟ-BHB	N/A	1.04	180	D-BHB	0.98	0.89	Y
Vanoverschelde et al. (1993)	NR, CO, PLA+	HA	Arginine-lysine solution	6	Arginine-lysine D-BHB	4.2	1.77	70	D-BHB	N/A	1.15	N
Veneman et al. (1994)	R, CO, PLA+	HA	Hypoglycemic clamp	13	Na-D/ʟ-BHB	2.1	1.04	360	D-BHB	1.91	1.77	Y
Webber et al. (1994)	R, CO, PLA+	HA	Insulin glucose clamp	10	Na-D/ʟ-BHB	3	5	120	D-BHB	4.2	N/A	N
Hasselbalch et al. (1996)	R, CO, PLA+	HA	N/A	8	Na-D/ʟ-BHB	N/A	2.25	210	D-BHB	2.16	N/A	N
Pan et al. (2001)	NR, 1A, PLA-	HA	N/A	6	Na-D/ʟ-BHB	4.2	1.04	75	D-BHB	2.78	2.43	Y
Blomqvist et al. (2002)	NR, 1A, PLA-	HA	N/A	6	D/ʟ-BHB	3	1.5	70	D-BHB	0.98	0.95	Y
		T1D	N/A	6	D/ʟ-BHB	3	1.5	70	D-BHB	1.27	1.26	Y
Pan et al. (2002)	NR, 1A, PLA-	HA	N/A	4	Na-D/ʟ-BHB	16.7^c^	2.29	120	D-BHB	2.25	N/A	N
Gormsen et al. (2017)	R, CO, PLA+	HA	Hyperinsulinemic euglycemic clamp	8	Na-D/ʟ-BHB	N/A	3*	390	totBHB	3.89	3.78	Y
Mikkelsen et al. (2015)	NR, CO, PLA-	HA	N/A	6	D-BHB	0	0.52	290	totBHB	0.37	0.36	Y
		HA	N/A	6	D-BHB	0	1.03	290	totBHB	0.68	0.63	Y
		HA	N/A	6	D-BHB	0	2.04	290	totBHB	1.66	1.58	Y
Svart et al. (2018)	R, CO, PLA+	HA	N/A	9	Na-D/ʟ-BHB	N/A	3.67*	240	totBHB	5.51	5.14	Y
Thomsen et al. (2018)	R, CO, PLA+	HA	LPS and Acipimox	10	Na-D/ʟ-BHB	N/A	2.43*	420	totBHB	3.45	N/A	N
Nielsen et al. (2019)	R, CO, PLA+	CHF	Low dose insulinemic euglycemic clamp	16	Na-D/ʟ-BHB	N/A	3*	180	totBHB	3.53	3.29	Y
		CHF	Low dose insulinemic euglycemic clamp	16	Na-D/ʟ-BHB	N/A	3*	180	totBHB	3.14	2.91	Y
		CHF	Low dose insulinemic euglycemic clamp	8	Na-D/ʟ-BHB	N/A	0.75*	180	totBHB	0.7	N/A	N
		CHF	Low dose insulinemic euglycemic clamp	8	Na-D/ʟ-BHB	N/A	1.5*	180	totBHB	1.6	N/A	N
Jensen et al. (2020)	R, CO, PLA+	T2D	Glucose clamp	18	Na-D/ʟ-BHB	N/A	1.84	165	D-BHB	2.5	2.4	Y

a ^4 mg/kg bolus.^

b ^13 mg/kg bolus.^

c ^16.7 mL/kg.^

*Infusion rate is for total BHB, not D-BHB. Abbreviations: 1A, single arm; D-BHB, D-beta-hydroxybutyrate; ʟ-BHB, ʟ-beta-hydroxybutyrate; CHF, chronic heart failure; Cmax, maximal concentration; Css, steady state concentration; CO, crossover; HA, healthy adults; IV, intravenous; N, no; N/A, not applicable; Na- D/L-BHB, racemic sodium beta-hydroxybutyrate, NR, non-randomized; PLA+, placebo controlled; PLA-, no placebo; R, randomized, T1D, type one diabetes; T2D, type 2 diabetes; totBHB, total beta-hydroxybutyrate; Y, yes.

**FIGURE 2:**
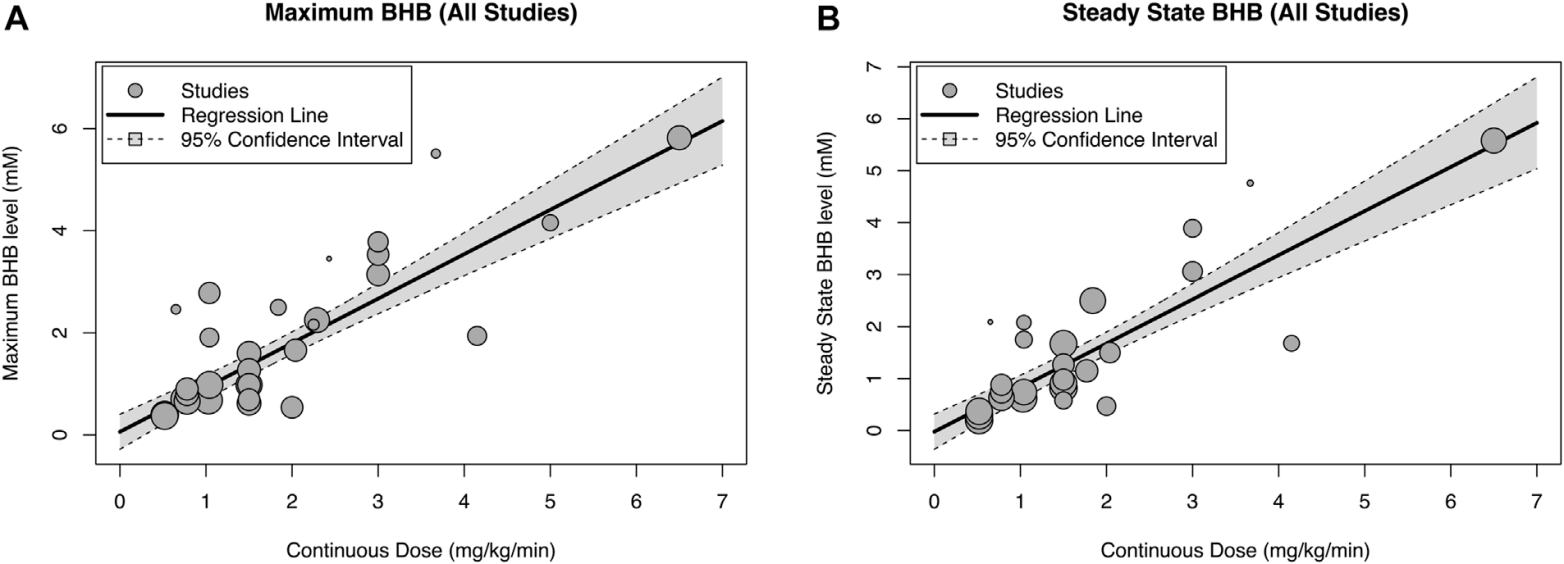
Relationship between blood beta-hydroxybutyrate (BHB) concentration and infusion rate extracted from all studies giving maximal [**(A)**, *n* = 31] and steady state [**(B)**, *n* = 26] concentration data. The diameter of the point for each study is proportional to the inverse of the variance.

There was a strong relationship between BHB infusion rate vs. BHB-C_max_ (y = 0.060 + 0.870x, *R* ^2^ = 87.2%, *p* < 0.0001, Figure 2A) and vs. BHB-C_ss_ (y = −0.022 + 0.849x, *R* ^2^ = 86.9%, *p* < 0.0001, Figure 2B). Increasing BHB infusion rate led to greater BHB concentrations in a linear manner within the studied concentration range. This relationship was maintained when a sensitivity analysis was performed to remove studies that did not present time course data (y = 0.135 + 0.863x, *R* ^2^ = 83.7%, *p* < 0.0001, Table 2), and to remove studies with non-healthy participants (y = 0.059 + 0.831x, *R* ^2^ = 96.3%, *p* < 0.0001, Table 2).

**TABLE 2:** Results of meta-regression analysis describing the primary analysis of all studies that reported maximal and steady state BHB concentrations, and sensitivity analysis which removed studies that did not report a time course, and removed studies that included disease populations. Abbreviations: BHB, beta-hydroxybutyrate; C_max_, maximal concentration; C_ss_, steady state concentration.

	Outcome (mM)	No. Studies	Equation	Tau	I ^ 2	R ^ 2	*p*-value
Primary Analysis							
1. BHB-C_max_		31	y = 0.060 + 0.870x	0.48	92.5%	87.2%	<0.0001
2. BHB-C_ss_		27	y = −0.022 + 0.849x	0.46	93.4%	86.9%	<0.0001
Sensitivity Analysis							
All Studies	3. BHB-C_max_ (Time Course Only)	23	y = 0.135 + 0.863x	0.58	95.5%	83.7%	<0.0001
Healthy Subjects Only	4. BHB-C_max_ (All Studies)	22	y = 0.059 + 0.831x	0.65	96.3%	81.8%	<0.0001
	5. BHB-C_max_ (Time Course Only)	16	y = 0.168 + 0.812x	0.74	98.0%	77.8%	<0.0001
	6. BHB-C_ss_	19	y = −0.237 + 0.867x	0.18	72.8%	98.3%	<0.0001

## 4 Discussion

The aim of the present study was to aggregate and standardize available data on the relationship between ketone delivery and blood concentration. As expected, we found a strong, linear relationship between IV BHB delivery rate and blood BHB within the ranges studied here, providing a useful regression that can guide the design of dosing regimens of ketone strategies in a research or clinical setting.

The main aim of this systematic review was to generate a robust, evidence-based regression, which could be used to estimate BHB delivery required to maintain a desired BHB-C_max_ or BHB-C_ss_ for therapeutic interventions (Table 3). As BHB can function as an alternative metabolic substrate to glucose during energetic stress, there is increasing interest in use of IV BHB in acute, in-patient conditions such as traumatic brain injury, or ischemic injury to the brain or heart (White et al., 2021). Furthermore, BHB has multiple signaling targets including HDAC inhibition, NLRP3 inhibition, activation of GPR41 and FFAR3 and direct post-translational modification of proteins [reviewed in (Puchalska and Crawford, 2021)], all of which may be activated at a different BHB concentration. Whilst the therapeutic window or threshold values for BHB required for different clinical endpoints [i.e., cardiac output (Nielsen et al., 2019), brain energy rescue (Fortier et al., 2021)] are not yet clearly defined, an important finding of this systematic review is that macronutrient-like amounts of BHB are required to maintain even relatively low blood BHB concentrations. The high delivery required will necessitate innovative delivery methods if BHB is to be used as a therapeutic agent.

**TABLE 3:** Guideline D-BHB salt infusion rates for a target plasma D-BHB C_ss_ using regression 2 (y = −0.022 + 0.849x).

Target D-BHB (mM)	D-BHB infusion rate (mg/kg/min)
0.5	0.56
1	1.15
2	2.33
4	4.87

A key problem addressed by this systematic review is the heterogeneity of the experimental and reporting methodologies used across the studies of IV BHB infusion. Firstly, comparisons between papers were not straightforward due to the range of units used to report BHB infusion rate and BHB concentration. Secondly and critically, very few papers explicitly highlighted that infusion of racemic BHB in conjunction with enzymatic measurement of only the D-BHB isoform, without adjustment of either value, leads to a gross overestimate of BHB delivery rate for a given blood D-BHB concentration. This is somewhat understandable as the alternatives to cheap, convenient D-BHB specific enzymatic methods are expensive, less accessible non-enzymatic methods that measure total BHB such as gas chromatography mass spectrometry. Use of pure D-BHB salts, or ketone esters that deliver only D- BHB and BHB precursors does not substantially elevate ʟ-BHB (Crabtree et al., 2023) and so adjustment is not required. We suggest that future exogenous ketone studies measure total BHB when a racemic mixture is administered or adjust the delivery rate or measured D-BHB concentration to account for the delivery of ʟ-BHB, that is, not captured in the concentration data.

IV infusions of ketone are translatable to an in-patient, acute setting but are not practical for chronic outpatient use. Therefore, a recent focus in keto-therapeutics is the development of orally ingestible ketones in the form of ketone salts, similar to those infused in the papers included here, or ketone esters (Stubbs et al., 2017; Puchalska and Crawford, 2021). The relationship described by our data for IV BHB infusions represents maximal BHB bioavailability for a given delivery rate. Any oral delivery strategy would be expected to result in some loss through lack of absorption or metabolism by enterocytes or other tissues prior to appearance in the systemic circulation. Svart et al. (2018) carried out a study aiming to match blood ketone concentrations delivered by oral and IV BHB salt delivery, and reported that 76% of the oral volume was required to match BHB concentrations via the IV delivery methods. However, nasogastric delivery of a ketone monoester at a primed-constant infusion rate of 3.2 mg/kg/min resulted in a BHB-C_ss_ of ∼2.8 mM; regression two based on our IV data would predict a similar BHB-C_ss_ of 2.7 mM. The relationship between BHB delivery and blood concentrations with oral and intravenous delivery requires further study to determine the adjustment required when comparing between the two delivery methods.

The conclusions that can be drawn from this analysis do have several important limitations. Firstly, whilst the relationship between BHB concentration and blood BHB was linear within the concentration ranges studied, this may not be the case at higher concentrations. The maximal concentration reported in this analysis was ∼6 mM. Given that in the seminal early studies of starvation ketosis undertaken by Cahill et al. (1966) ketosis plateaued at around 6 mM after 25 days of starvation, concentrations above this are not likely relevant to health outcomes in a “physiological” context (Krebs, 1966). However, higher concentrations are seen during pathological states of ketoacidosis (Laffel, 1999), so it is important to note that the delivery-concentration relationship outlined in this systematic review is unlikely to be relevant to ketosis seen during metabolic crisis. Secondly, we adjusted the infusion rate of BHB to be 50% lower when racemic BHB was infused. This is based on the assumption that all racemic salts used in the studies were 50/50 of each BHB isoform; however, this was not confirmed analytically in any of the studies included. Thirdly, as the majority of the studies were conducted using racemic salts and circulating ʟ-BHB concentrations were not measured, it is unknown if and how the presence of ʟ-BHB could have affected metabolism of D-BHB, which may have changed the relationship between delivery and concentration vs. if only D-BHB was infused. Whilst a major metabolic fate of ʟ-BHB is thought to be conversion to D-BHB (Desrochers et al., 1992), some ʟ-BHB may also be converted to fatty acids and sterols (Desrochers et al., 1992). Additionally, whilst ketone disappearance is largely expected to occur due to oxidation, some ketone removal may be accounted for by excretion in urine and breath, or temporary sequestration tissue prior to oxidation. In order to understand the relationship between BHB concentration and different metabolic fates, tracer labelling studies would be necessary. Finally, it is important to note that other variables in subject populations might alter BHB metabolism, such as feeding state, habitual diet or exercise training status.

In summary, this systematic review has standardized and compiled the literature on IV BHB infusions in adults and demonstrates that BHB concentrations are related to IV BHB delivery in a linear manner up to ∼6 mM of BHB. The regression that resulted from the systematic review can be used to predict the IV BHB delivery rate and amount required to achieve a targeted BHB concentration in research studies and possibly for future clinical applications of IV BHB.

## Data Availability

The original contributions presented in the study are included in the article/Supplementary Material, further inquiries can be directed to the corresponding author.
